# Transcription and splicing regulation in human umbilical vein endothelial cells under hypoxic stress conditions by exon array

**DOI:** 10.1186/1471-2164-10-126

**Published:** 2009-03-25

**Authors:** Xingyi Hang, Peiyao Li, Zhifeng Li, Wubin Qu, Ying Yu, Hualing Li, Zhiyong Shen, Hao Zheng, Yan Gao, Yonghong Wu, Minghua Deng, Zhixian Sun, Chenggang Zhang

**Affiliations:** 1 Beijing Institute of Radiation Medicine, State Key Laboratory of Proteomics, Beijing 100850, PR China; 2 LMAM, School of Mathematical Sciences and Center for Theoretical Biology, Peking University, Beijing 100871, PR China

## Abstract

**Background:**

The balance between endothelial cell survival and apoptosis during stress is an important cellular process for vessel integrity and vascular homeostasis, and it is also pivotal in angiogenesis during the development of many vascular diseases. However, the underlying molecular mechanisms remain largely unknown. Although both transcription and alternative splicing are important in regulating gene expression in endothelial cells under stress, the regulatory mechanisms underlying this state and their interactions have not yet been studied on a genome-wide basis.

**Results:**

Human umbilical vein endothelial cells (HUVECs) were treated with cobalt chloride (CoCl_2_) both to mimic hypoxia and to induce cell apoptosis and alternative splicing responses. Cell apoptosis rate analysis indicated that HUVECs exposed to 300 μM CoCl_2 _for 24 hrs were initially counterbalancing apoptosis with cell survival. We therefore used the Affymetrix exon array system to determine genome-wide transcript- and exon-level differential expression. Other than 1583 differentially expressed transcripts, 342 alternatively spliced exons were detected and classified by different splicing types. Sixteen alternatively spliced exons were validated by RT-PCR. Furthermore, direct evidence for the ongoing balance between HUVEC survival and apoptosis was provided by Gene Ontology (GO) and protein function, as well as protein domain and pathway enrichment analyses of the differentially expressed transcripts. Importantly, a novel molecular module, in which the heat shock protein (HSP) families play a significant role, was found to be activated under mimicked hypoxia conditions. In addition, 46% of the transcripts containing stress-modulated exons were differentially expressed, indicating the possibility of combinatorial regulation of transcription and splicing.

**Conclusion:**

The exon array system effectively profiles gene expression and splicing on the genome-wide scale. Based on this approach, our data suggest that transcription and splicing not only regulate gene expression, but also carry out combinational regulation of the balance between survival and apoptosis of HUVECs under mimicked hypoxia conditions. Since cell survival following the apoptotic challenge is pivotal in angiogenesis during the development of many vascular diseases, our results may advance the knowledge of multilevel gene regulation in endothelial cells under physiological and pathological conditions.

## Background

The balance between endothelial cell (EC) survival and apoptosis is an important cellular process involved in preserving blood vessel integrity and vascular homeostasis [1-4]. Lining the surface of vascular structures, ECs should endure a variety of normal or abnormal stresses that are both chemical and physical in nature. Aberrant stresses may break the dynamic balance and contribute to irreversible endothelial dysfunctions due to EC apoptosis and vessel integrity defects [4-6]. Studies have demonstrated that modulating this balance is important in the initiation and development of many vascular diseases, *e.g*. stroke, diabetic retinopathies, thrombosis, and atherosclerosis [2,7-9]. Therefore, identifying the regulatory mechanisms of the survival and apoptosis of ECs may provide opportunities to improve clinical therapies for the treatment of these vascular diseases.

Transcription has been well studied and has been shown to be of considerable importance in modulating EC apoptosis [10,11]. Alternative splicing (AS), an important molecular mechanism increasing proteome diversity via the assembly of different exons, has been reported to regulate cellular processes in endothelial systems under stress. For example, a splicing isoform of platelet endothelial cell adhesion molecule-1 (*PECAM-1*, a suppressor of cell apoptosis) was proven to activate the EPH receptor B2 (*EPHB2*) in response to the early stages of shear stress [12]. Splicing variants of vascular endothelial growth factor (*VEGF*) provide a balance of pro- and anti-angiogenic regulation, and they also act as determinants of tumor angiogenesis [13]. Importantly, one study has reported that AS, like transcription, can enable rapid and specific changes in gene expression in response to stress [14]. Thus, elucidating the transcriptional and splicing regulation that affects EC survival and apoptosis is critical for a better understanding of endothelial function under physiological and pathological stresses.

Although many studies have focused on transcriptional and proteome profiling of ECs under stress [15,16], no study to date has addressed splicing and multilevel regulation from a genomic standpoint. Here, human umbilical vein endothelial cells (HUVECs) were treated with 300 μM CoCl_2 _for 24 hrs to mimic hypoxia [17-19] and to induce cell apoptosis and alternative splicing responses, as previously described [20,21]. An Affymetrix Human Exon 1.0 ST array system containing over 1 million exon clusters and 5.5 million features was used to profile gene expression at both the transcriptional and splicing levels. After a comparative analysis of expression between treated and normal samples, Gene Ontology (GO) and protein annotation coupled with pathway analysis provided evidence illustrating the balance between cell survival and apoptosis. Furthermore, the classification of splicing patterns and the discovery of a group of genes affected by both transcription and splicing indicated multilevel regulations representing the response of HUVECs to stress. Our data may facilitate the development of new therapeutic approaches for vascular disease treatment.

## Results

### Analysis of apoptosis in CoCl_2_-treated HUVECs

**Figure 1:**
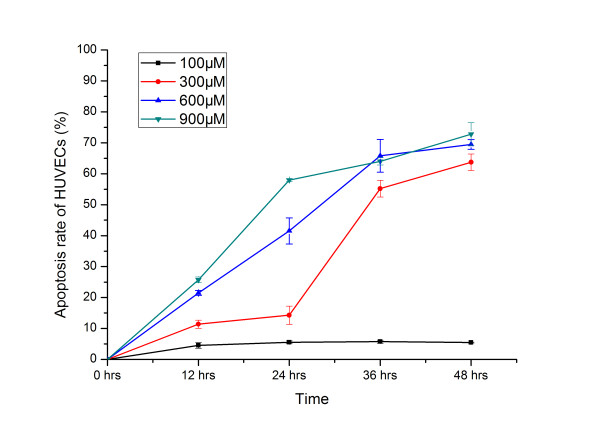
**Apoptosis rate analysis of the HUVECs with CoCl_2 _treatment**. HUVECs were incubated with various concentrations of CoCl_2 _(0, 100, 300, 600 and 900 μM) for 0, 12, 24, 36 and 48 hrs. The cell apoptosis rate at each time point was assayed using a flow cytometry assay. The following RNA sources were used for amplification: Normal (normal cultural HUVECs) and CoCl_2 _(300 μM CoCl_2_-treated HUVECs).

To mimic hypoxia stress, HUVECs were incubated with 100, 300, 600 and 900 μM CoCl_2 _for 0 (control), 12, 24, 36 and 48 hrs. The apoptosis rate of HUVECs treated with different concentrations of CoCl_2 _was analyzed by flow cytometry (See Figure 1). The apoptosis rate rapidly increased as the CoCl_2 _concentrations and incubation time increased, although the increase did not occur in a linear fashion. Clearly, 100 μM CoCl_2 _had a minimal effect, as evidenced by persistently low apoptosis rates over time, while 600 μM and 900 μM CoCl_2 _induced high rates of cellular apoptosis earlier. When incubated with 300 μM CoCl_2_, HUVECs showed a large transition in apoptosis rates, which increased from 14% to 55% between 24 and 36 hrs. We therefore considered the first 24 hrs of HUVECs with 300 μM CoCl_2 _treatment as the early stage of apoptosis.

### Differentially expressed genes and functional analysis

A comparison of the mimicked hypoxic and normoxic groups identified 1583 differentially expressed genes (DEGs), consisting of 300 (19%) upregulated and 1283 (81%) downregulated genes (See Additional file 1). The number of downregulated genes was 4.28 (1283/300) times higher than the number of upregulated genes in response to stress. A different strategy of functional analysis (other than normal GO analysis) was performed on DEGs. First, function enrichments of DEGs were detected based on their protein annotations from the UniProt database [22]. Interestingly, 24% of the DEGs were categorized as genes undergoing or regulating alternative splicing (See Table 1). It is equally notable that the other functional categories available for short-term cellular response to hypoxia include nuclear protein, phosphorylation, metal binding, and DNA-binding, which are also prominent in enrichment (See Table 1). These categories demonstrate extensive responses of gene regulation to hypoxia. GO analysis was then carried out on the up- and downregulated genes respectively. Importantly, we found that "programmed cell death" (Fisher's exact test, *P *= 2.1 × 10^-7^) is only significantly observed in the upregulated genes, which indicates that apoptosis is initiated in response to mimicked hypoxia in HUVECs.

**Table 1:** List of the top eight functional categories of DEGs, based on the protein annotations of the UniProt databases

Rank	Terms	Count	%	*P*-value
1	Alternative splicing	381	24.1	1.3E-23
2	Nuclear protein	355	22.4	1.2E-29
3	Phosphorylation	312	19.7	3.0E-58
4	Metal-binding	191	12.1	8.8E-5
5	Nucleotide-binding	190	12.0	1.4E-23
6	Transferase	164	10.3	2.6E-16
7	ATP-binding	157	9.9	5.4E-22
8	DNA-binding	137	8.7	5.1E-5

(Threshold of significance: *P *< 1 × 10^-4^, Fisher's exact test)

### Alternative splicing events and protein domain analysis

Using the "Splicing Index" algorithm described in the methods, 342 probe select regions (PSRs) labeled as "core" were identified as alternatively spliced exons (See Additional file 2), and these belonged to 293 alternatively spliced transcripts. Compared with normal HUVECs, 250 PSRs with higher expression were classified as "general exon inclusion" events, while the other 92 PSRs with lower expression were considered as "general exon skipping" events. Thirty-five percent (102/293) of the alternatively spliced transcripts are supported by experimental evidence based on the NCBI RefSeq database records. Since it is possible for multiple alternative splicing events to occur in the same transcript, 13% (37/293) of the transcripts were found to contain 25% (86/342) of the alternatively spliced exons. Therefore, there was an average of 2.3 (86/37) alternatively spliced exons per transcript under mimicked hypoxia conditions in our data. A typical example is ubiquitin-associated protein 2 (*UBAP2*), for which five alternatively spliced exons were detected, indicating a complicated pattern of splicing regulation of *UBAP2 *in HUVECs under hypoxic stress.

It is well known that splicing not only provides feedback to affect transcription, but also feeds forward to modulate protein function. An InterProScan search revealed 105 protein domains in 21% (71/342) of the coding regions of alternatively spliced exons, and 70 different domains were revealed after removing the redundant records (See Additional file 3).

### RT-PCR and quantitative Real-time PCR validation

**Figure 2:**
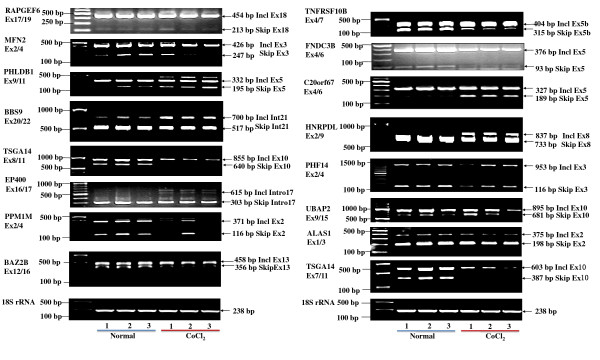
**RT-PCR validation of splicing events identified by exon array**. PCR products were derived using forward/reverse primers to amplify the flanking regions indicated under the gene name. The mobility changes are identified on the right side ("Incl" for exon inclusion; "Skip" for exon skipping). The 18S rRNA was used as a control. The primer sequences are available in an online Excel table (See Additional file 5).

**Figure 3:**
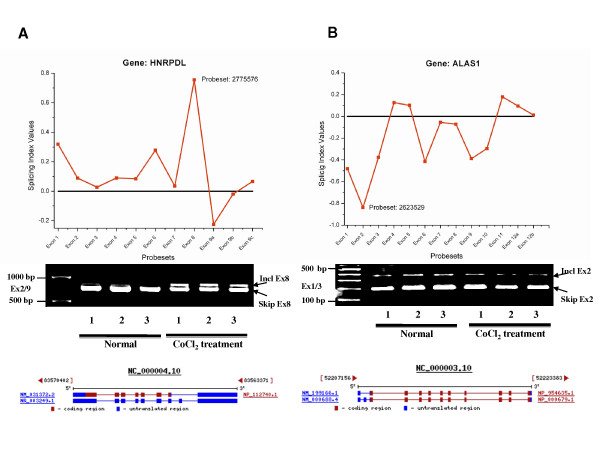
**Consistency among results of exon array, RT-PCR and isoform evidence from RefSeq database**. The "Splicing Index" value measures a quantitative change in the exon expression between mimicked hypoxia and normoxia samples. A: The *HNRPDL *gene, mimicked hypoxia-specific "Splicing Index" values illustrated for isoform supported exons and RT-PCR validation using primers located in flanking exons. B: The *ALAS1 *gene, illustrated as in A.

In order to confirm the DEGs detected by exon array, 14 DEGs exhibiting highly significant differences in expression or with important functions (for example, splicing factors) were validated by RT-PCR in Figure S1 (See Additional file 4). Interleukin 8 (*IL-8*) and hypoxia-inducible factor 1 (*HIF-1α*), two important genes in hypoxia response, were validated by real-time quantitative PCR (RT-qPCR). Consistency was found between the results obtained by RT-qPCR and those acquired by the exon array system (See Table 2). Thirty-two differentially expressed exons were selected for validation by RT-PCR. Forward and reverse PCR primers were designed adjacent to or spanning several constitutive exons (See Additional file 5), and half of these primers amplified specific bands of differentially expressed transcripts (See Figure 2). Furthermore, two genes, *HNRPDL *(a splicing factor) and *ALAS1 *(a kind of synthase), are shown in Figure 3 to compare the results of the exon array system, RT-PCR, and RefSeq isoform evidence for consistency. Positive and negative values of "Splicing Index" indicate the "exon inclusion" and "exon skipping" events, respectively, in mimicked hypoxia samples compared with controls. In Figure 3A, exon 8 of the *HNRPDL *gene is highly included in the condition of mimicked hypoxia, which is consistent with the results of the RT-PCR and RefSeq isoforms. The situation is true for skipping of exon 2 of *ALAS1 *gene shown in Figure 3B. All these results suggest that the exon array system is reliable and effective enough to detect differential expression at both the transcriptional and splicing levels.

**Table 2:** Relative quantitative comparison between the RT-qPCR technique and exon array system

	**Time**	**Gene average C_T_**	**GAPDH average C_T_**	**ΔC_T_** **Gene- GAPDH**	**ΔΔC_T_, ΔC_T_-ΔC_T, 0 h_**	**Fold change to 0 h^a^**	**Fold change** **based on exon array system^b^**
**HIF-1α**	0 h	18.84 ± 0.47	16.67 ± 0.23	18.84 ± 0.47	0.00 ± 0.47	1.00 (0.72~1.39)	1
	24 hrs	20.77 ± 0.37	15.33 ± 0.30	20.77 ± 0.37	1.93 ± 0.37	0.26 (0.20~0.34)	0.18
**IL-8**	0 h	21.78 ± 0.33	16.67 ± 0.23	5.12 ± 0.41	0.00 ± 0.41	1.00 (0.75~1.32)	1
	24 hrs	18.15 ± 0.09	15.33 ± 0.30	2.82 ± 0.31	-2.30 ± 0.31	4.92 (4.03~6.11)	7.66

^a^Fold change based on RT-qPCR is calculated as 2^-ΔΔCT^

^b^Fold change based on exon array is from samr d scores for gene expression

### Analysis of transcription and splicing pathways

**Figure 4:**
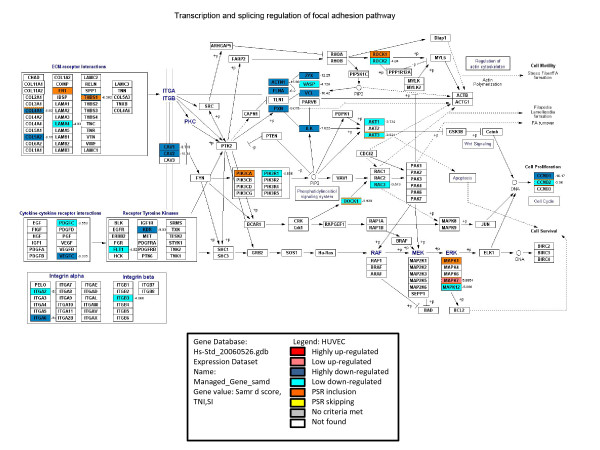
**Mapping of differentially expressed genes and exons to "Focal Adhesion" pathway**. The magnitudes of gene expression differences are indicated by samr d scores, while the "TNS" rate and "Splicing Index" *P*-values indicate the magnitudes of the differences of exons expression and their statistical significance, respectively. GenMAPP displays colors within each gene box based on these values (red for upregulated, blue for downregulated, orange for activated exons (PSRs), and yellow for skipped exons (PSRs)). GenMAPP prioritizes the central and rim color assignments for gene boxes based on the order of the underlying data.

The KeggChart tool in the DAVID system were used to detect pathways enriched in up- and downregulated genes based on the KEGG database. As shown in Table 3, the "MAPK signaling pathway" and "Proteasome" were highly activated, while "Focal adhesion" and "Regulation of actin cytoskeleton" were largely silenced. However, there was no significant enrichment for alternatively spliced genes in the KEGG pathways. We therefore used GenMAPP to map both DEGs and alternatively spliced genes simultaneously based on the context of the KEGG pathways. Interestingly, we found that the "Focal Adhesion" pathway contained not only 37 downregulated genes, but also 9 exon inclusion events. Genes affected at both the transcription and splicing levels appeared in the "Focal Adhesion" pathway. Five of these genes were simultaneously regulated at both levels (See Figure 4).

**Table 3:** Pathway enrichment of the up- and downregulated genes based on the KEGG database analysis

Term	Count	%	*P*-value
Upregulated pathways			
HSA04010: MAPK signaling pathway	11	3.70%	7.4E-03
HSA03050: Proteasome	7	2.36%	1.0E-05
HSA04612: Antigen processing and presentation	6	2.02%	6.2E-03
Downregulated pathways			
HSA04510: Focal adhesion	37	2.90%	5.4E-05
HSA04810: Regulation of actin cytoskeleton	30	2.35%	6.4E-03
HSA04110: Cell cycle	29	2.27%	1.2E-07
HSA00240: Pyrimidine metabolism	18	1.41%	2.9E-03
HSA04350: TGF-beta signaling pathway	17	1.33%	2.1E-03

**Figure 5:**
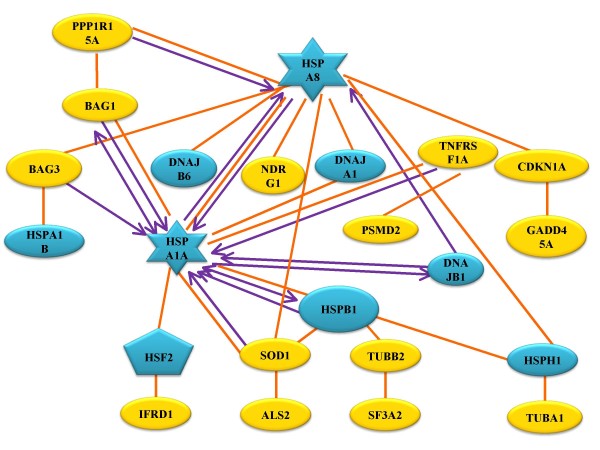
**Illustration of the activated module of heat shock proteins in HUVEC response to stress**. Elliptical nodes represent general genes, while star-like nodes represent hub genes in the scale-free module. Blue nodes represent heat shock proteins, while yellow nodes represent non-heat shock proteins. Orange lines designate binding and interaction, and purple lines designate directed regulations, such as transcription, signal transduction, *etc*. The arrows represent the direction of regulation.

Furthermore, a two-step literature mining strategy was carried out to explore the functional modules from biological networks for the HUVECs under mimicked hypoxia conditions. A novel schematic molecular module was generated to illustrate functional modularity within networks (See Figure 5). This module contained 23 proteins and 40 regulatory relationships (See Additional file 6), of which 8 heat shock proteins and one heat shock transcription factor were upregulated, indicating that these heat shock proteins may function synergistically in this module in response to hypoxic stress.

### Splicing patterns and complex regulation between transcription and splicing

**Figure 6:**
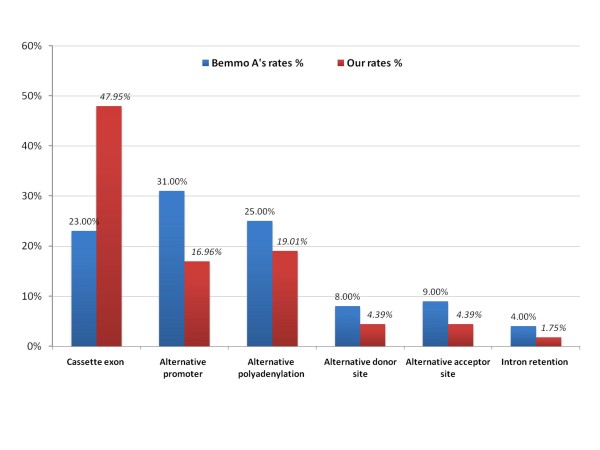
**Comparison of splicing pattern distribution between Bemmo's data and ours**. Bemmo A *et al*. used the MAQC human tissue samples to obtain an overall estimation of splicing pattern distributions [26]. We selected the data produced by the "Splicing Index" algorithm, which is the most similar to our approach. The labels on the histograms are percentages of the splicing patterns for alternative splicing events. It can be seen that the distribution of splicing patterns observed in the affected HUVECs varies with the selected background distribution.

In the distribution of splicing patterns, 48% of AS events were considered to be of the "cassette exon" type, which is consistent with another report that "cassette exon" is a kind of splicing pattern with high frequency [23]. The "alternative promoter" category comprised 17% of the AS events. Previous studies have reported that alternative promoters can regulate at both the transcription and splicing levels [24,25]. Comparing with a benchmarked exon array data that were also analyzed by the "Splicing Index" algorithm, we found that the proportions of the splicing patterns observed in HUVECs treated with CoCl_2 _are different from those in the benchmark dataset [26]. The proportion of "cassette exon" is nearly double, while those of all the others decrease (See Figure 6). Other than the technical differences between the experiments, we hypothesize that the proportion of the splicing patterns may be specific to different phenotypic conditions. For example, the "cassette exon" is more dominant in the stress-induced HUVECs than in the benchmarked exon array data. By classifying all AS events into "general exon inclusion" events (upregulated exons) and "general exon skipping" events (downregulated exons) on the basis of exon expression levels, we surprisingly found that the "general exon inclusion" events are highly correlated with the downregulation of the genes, while "general exon skipping" events are highly correlated with the upregulation of the genes (Fisher's exact test. *P *= 2.2 × 10^-16^).

Furthermore, we found that a large proportion of alternatively spliced genes overlapped with DEGs (46% of alternatively spliced genes, 134/293), and the overlapping genes differentially expressed at both the gene and exon levels. Afterwards, a functional analysis similar to Table 1 was performed on the alternatively spliced genes. Interestingly, parallel rankings of functional categories were found between alternatively spliced genes and DEGs because of the high proportion of overlaps between these two gene sets. The top three categories of alternatively spliced genes ("alternative splicing" with *P *= 2.5 × 10^-13^, "nuclear protein" with *P *= 5.0 × 10^-7^, and "phosphorylation" with *P *= 1.3 × 10^-15^, all Fisher's exact test) were very consistent with their counterparts at the gene level. We also found that alternatively spliced genes with multiple affected exons were largely included among the overlapping genes (54%, *P *= 2.9 × 10^-53^, Chi-square test). Finally, 17 alternatively spliced genes (See Additional file 7)were transcription factors supported by the publicly available TRANSFAC 7.0 database (Fisher's exact test, *P*-value = 2.6 × 10^-12^).

## Discussion

### Functional and pathway analyses support the conflicting balance between HUVEC survival and apoptosis

Consistent with the results of cell apoptosis analysis, the gene function and pathway analyses of DEGs revealed the conflicting balance between HUVEC survival and apoptosis. *IL-8*, a gene known to directly promote endothelial cell survival and angiogenesis, has been demonstrated to be highly upregulated based on exon array system and RT-qPCR analysis [27]. This suggests that chemokines may play an important role in resisting apoptosis in HUVECs. HUVEC survival may decrease with the expression of thrombospondin-1 (*THBS1*), which has been reported to induce endothelial cell apoptosis and inhibit angiogenesis [28]. On the other hand, Yang et al. reported that loss of survivin (*BIRC5*) increased cellular sensitivity to apoptotic stimuli and caused spontaneous apoptosis [29]. Our results indicated that downregulation of survivin in HUVECs is highly likely to result in apoptosis via this mechanism. It has also been reported that *AIFM2 *reduces cell survival signaling and contributes to the onset of apoptosis [30]. The observed upregulation of *AIFM2 *suggests that this gene also plays a role in promoting cell apoptosis. These gene expression patterns indicated that HUVECs struggle to avoid apoptosis in order to survive under stress. In the results of the GO analysis, it is notable that the upregulated genes are significantly enriched in the "programmed cell death" functional annotation, demonstrating the ongoing apoptosis of HUVECs.

Since genes are usually functionally organized into pathways, it is necessary to explore the gene regulation in terms of the pathways involved. As shown in Table 3, the "Focal Adhesion" pathway is largely silenced, which is congruous with the fact that adhesion-dependent endothelial cell survival is regulated by focal adhesion kinase [31]. This silenced pathway may result in the disorder of the cellular signaling that mediates the contact between endothelial cells and the extracellular matrix during apoptosis [32]. In addition, Kulms et al. showed that disruption of the "Actin cytoskeleton" (in the downregulated pathway) is mediated via the activation of *CD95 *(*Fas/APO-1*) during the induction of apoptosis [33]. With regard to the upregulated pathways, the "MAPK signaling" pathway was studied by inducing apoptosis in endothelial cells via phosphorylation [34]. The upregulated "Antigen processing and presentation" pathway is supported by the expression of many antigens, especially platelet endothelial cell adhesion molecule-1 (*PECAM-1*/*CD31 *antigen), which provides survival signals to suppress apoptosis [35]. However, the regulation of the "Proteasome" pathway is somewhat complex because proteasome inhibitors have dual functions, either facilitating or inhibiting apoptosis [36]. In conclusion, the expression of genes in the examined pathways presents a comprehensive illustration of the state of homeostasis between cell survival and apoptosis.

Finally, a novel heat shock protein module composed of the Hsp27, Hsp70, Hsp105, and DnaJ subfamilies was discovered to underlie the functional modulation of biological networks under stress. These heat shock proteins have been individually demonstrated to resist apoptosis in response to a variety of stimuli including hypoxia [37-40]. Figure 5 shows that the 70 kDa heat shock protein 1A (HSPA1A) may function together with other heat shock proteins to form a protein complex that more effectively inhibits apoptosis. Notably, HSPA1A has been reported to confer resistance to apoptosis in conjunction with other heat shock proteins [41], which is consistent with its feature as a hub of the scale-free module. This novel module suggests that heat shock proteins and their collective regulation may be crucial to controlling HUVEC survival and apoptosis.

### Complex regulation of transcription and splicing in stress induced HUVECs

Both dependent and independent regulations of transcription and splicing usually coexist under most physiological and pathological conditions. Based on the observation of a higher rate of overlapping between DEGs and alternatively spliced genes than that found in other studies [42,43], we expect the possibility of combinatorial regulation between transcription and splicing in stress induced HUVECs. Although we also found that the general splicing patterns are highly correlated with gene expression levels, the exact molecular mechanism of the coupling regulation is still unknown. We hypothesize that splicing may modify the transcription activity or RNA stability [44,45], while transcription may change the splicing efficiency [46]. On one hand, alternative splicing of mRNA can change RNA stability, which in turn will probably affect the expression levels of the gene transcripts with different RNA stability [47]. On the other hand, it is also possible that different expression levels of the upstream genes of splicing factors facilitate or inhibit splicing machinery by influencing spliceosome assembly or the *cis*-elements during the splicing process. These two aspects of regulations could both possibly result in the high degree of correlation between splicing patterns and transcriptional expression. Therefore, it is reasonable to speculate that HUVECs may utilize the combinatorial regulation of transcription and splicing to modulate the cellular response to stress finely and efficiently.

Transcription and splicing may be independent processes, but there are still possible correlations at specific spatiotemporal stages of the cellular response. In our results, 17 differentially expressed transcription factors (See Additional file 7) were detected as alternatively spliced genes(Fisher's exact test, *P*-value = 2.6 × 10^-12^). On the other hand, 15 splicing factors (See Additional file 7), including 6 SR proteins and 9 hnRNP proteins, were detected as DEGs (Fisher's exact test, *P*-value = 1.3 × 10^-6^). The existence of two possible regulatory mechanisms for these transcription factors and splicing factors can be conjectured: 1) the 17 alternatively spliced transcription factors are possible targets of splicing factors; 2) the 15 differentially expressed splicing factors are possible targets of transcription factors. If the differential expression of splicing factors directly influences the splicing efficiency and in turn triggers the alternative splicing of transcription factors, a loop of feedback regulation can then be established in response to stress. Since it is difficult to reveal the exact regulatory mechanisms underlying these *trans*-factors and their targets, further studies are needed to explain the regulatory model of the complex regulation under stress in the future.

### Alternative splicing can influence biological networks through domain architectures

Since no significant enrichment of alternatively spliced genes was found in the KEGG pathways, splicing may follow a different set of regulatory rules than transcription in pathways. Alternative splicing can expand the protein repertoire and influence protein function by altering protein domains. Melissa et al. reported that 7,179 of 22,218 human genes in the Ensembl database encoded two or more different proteins. Of these, 2,229 genes encoded proteins with different PFAM domain architectures [48]. The affected domains in the coding regions of alternatively spliced exons confirmed the existence of changes in the transcriptome and proteome resulting from alterations in the domain architecture of biological networks [49]. We found that alternative splicing may influence transcription through the gain or loss of promoter binding domains. For example, the number of zinc finger domains (IPR007087) decreased in zinc finger protein 589 (*ZNF589*), whose transcription factor activity depends on the number of domain repeats. The same phenomenon was also found in the WD-40 repeat domain (IPR001680) of the *SH3KBP1 *and *RRP9 *genes. In our results, the DNA-binding domain HMG-I(Y) (IPR000637) was lost in the high mobility group AT-hook 2 (*HMGA2*). Previous studies have demonstrated that the domain HMG-I(Y) functions as part of a hypoxia-induced enhanceosome, promoting the transcription of *COX-2 *in HUVECs [50]. Defects in the HMG-I(Y) DNA-binding domain will disorganize the transcriptional regulation under stress. The MAM domain (IPR000998) in neuropilin 1 (*NRP1*), representing adhesive function, may be altered to induce endothelial dysfunction in response to stress. These changes of domains were analyzed based on the coding regions of alternatively spliced exons (See Additional file 3).

## Conclusion

In this study, HUVECs were incubated with 300 μM CoCl_2 _for 24 hrs to induce the balance between cell survival and apoptosis, followed by a genome-wide expression profiling of transcription and splicing by exon array system. Functional and pathway analyses of gene levels and exon levels demonstrated the importance of transcription and splicing regulation in cellular processes. Evidence from the splicing classifications and the overlap between the two levels suggested a combinatorial regulation. Because very few studies have investigated splicing regulation in endothelial cell survival and apoptosis, elucidating the underlying mechanisms associated with these phenomena is critical for a better understanding of vascular biology under normal and pathological conditions.

## Methods

### Cell culture and cell apoptosis analysis

HUVECs were purchased from Cascade Biologics (USA) and cultured in Medium 200 supplemented with Low Serum Growth Supplement (LSGS) (Cascade Biologics, USA) in a CO_2 _incubator (5% CO_2_) at 37°C. The cells were treated with different concentrations of CoCl_2 _(100 μM, 300 μM, 600 μM, 900 μM) (Sigma, USA) for 0, 12, 24, 36 and 48 hrs to mimic hypoxia. The cells were then incubated with fluorescein isothiocyanate-conjugated Annexin V (A-FITC) and propidium iodide (PI) using the Apoptest kit (Jiancheng, China) according to the manufacturer's instructions. Flow cytometry analysis was performed using the FACSCalibur system (Becton Dickinson, USA). The data were analyzed using CellQuest software to estimate the apoptosis rate at different time points.

### Sample preparation and array hybridization

After being cultured under normoxia or mimicked hypoxia (300 μM CoCl_2 _for 24 hrs), total RNA was extracted from the HUVECs using the TRIzol reagent, according to the manufacturer's protocol (Invitrogen, USA). Total RNA was dissolved in an appropriate volume of DEPC-treated water following A_260_/A_280 _measurement, while the total RNA integrity was evaluated by electrophoresis in a denaturing gel. The RNA samples were further purified using DNase (TaKaRa, Japan). For each experimental condition, three independent replicate samples were obtained for exon array analysis. For each sample, 1 μg of RNA was processed using the Affymetrix GeneChip^® ^Whole Transcript Sense Target Labeling Assay. The GeneChip^® ^WT cDNA Synthesis Kit, the WT cDNA Amplification Kit, and the WT Terminal Labeling Kit (Affymetrix, Inc., Santa Clara, CA) were used for the sample preparation. 8 μg of cDNA were used for the second cycle cDNA reaction. Hybridization cocktails containing 3–4 μg of fragmented, end-labeled cDNA were applied to the GeneChip^® ^Human Exon 1.0 ST arrays. Hybridization was performed for 16 hrs using the MES_EukGE-WS2v5_450-DEV fluidics wash and stain script. The arrays were scanned using the Affymetrix GCS 3000 7G and Gene-Chip Operating Software v1.3 to produce the intensity files.

### RT-PCR and quantitative Real-time RT-PCR

1 μg of each RNA sample was used for first strand cDNA synthesis using SuperScript II reverse transcriptase (Invitrogen, USA) and a combination of random hexamer primers and oligo-dT in a total volume of 10 μl. PCR was carried out using 2 μl of cDNA, with specific primers flanking the constitutive exons, and ExTaq Polymerase (TaKaRa, Japan) in a volume of 25 μl. The conditions for PCR amplification were denaturation at 95°C for 5 min, 32 cycles of 95°C for 30 sec, 55°C for 30 sec, and 72°C for 45 sec, followed by a final elongation step at 72°C for 7 min. The PCR products were then separated on 1.5% agarose gels. The RT-PCR products were gel-purified using a PCR purification kit (Promega, USA) and subcloned into the pGEM-T Easy Vector (Promega, USA) for direct sequencing to validate the transcript variants.

1 μl of each cDNA product was used for quantitative real-time PCR amplification with SYBR Green PCR Master Mix (TianGen). The primers were designed and verified by the primer specificity-checking program MFEprimer [51]. PCR was carried out with an iCycler Real-time PCR detection system (Bio-Rad) under the following conditions (40 cycles): 95°C for 2 min, 95°C for 30 sec, 57°C for 30 sec, and 68°C for 30 sec. SYBR Green analyses were followed by dissociation curves in a temperature range of 60°C~90°C to assess the amplification specificity. Each sample was tested in triplicate and quantified according to the mean expression values obtained for both samples.

### Low level analysis of the exon array

Low-level analysis of the optical intensity files of the exon array (".CEL" format) was performed by Affymetrix Power Tools (APT). Background noise was detected by the "Detection above Background (DABG)" algorithm. Normalization was performed using the "quantile normalization" algorithm for both the exon and gene levels. The "Probe Logarithmic Intensity Error Estimation" (PLIER) algorithm was used to estimate exon signals based on probe intensities. At the gene level, a variant algorithm called "Iter-PLIER" was used to summarize gene signals from probeset intensities. The "Iter-PLIER" algorithm can discard probesets with inconsistent signals to avoid low-weighted effects introduced by differentially expressed exons.

### Filtering

Hierarchical filtering was then performed to eliminate noise and outliers at both the gene and exon levels. At the exon level, only the probesets considered "Present" (DABG *P *< 0.05) in at least 50% of the samples in either group were reserved. At the gene level, only the "core" meta-probesets with high confidence were used to estimate gene signals. The differentially expressed genes were considered acceptable based on two principles. First, genes with more than 50% of the "core" exons designated as "Present" (DABG *P *< 0.05) should appear in more than 50% of the samples in both groups. Second, the gene signals needed to exceed 100. We subsequently removed the probesets labeled as potential cross-hybridization targets based on Affymetrix CSV annotation files.

### Detection of differentially expressed genes and alternative splicing

A Bioconductor package called "samr" was used to infer the differentially expressed genes (DEGs) between mimicked hypoxia and normal groups. Corrections for multiple hypothesis testing included using the Benjamini-Hochberg method [52]. We set parameters Δ = 2.3 and FDR < 2.6 × 10^-4 ^as cut-off values for DEGs. Other than some regression models [53,54], most of the previously published papers used the "Splicing Index" model [55,56] to detect alternative splicing events from the exon array data. A program built in-house based on the "Splicing Index" model was used to detect differentially expressed exons. The rate of exon signals to summarized gene signals were defined as the transcription normalized exon signals:



The "Splicing Index (SI)" model was then employed to indicate alternative splicing capability based on the relative inclusion rate of exons:



The absolute value of SI represented the magnitude of difference of the exon inclusion rate between the two groups. To identify the significant alternatively spliced exons, a Student's *t*-test was used to compare TNS values between the two groups. Finally, the high proportion of true positives, with *P*-value < 0.015 and fold change magnitudes > 0.5, were retained as potential alternatively spliced exons.

### Data deposition

The raw ".CEL" files and normalized data at both the gene and exon levels have been deposited in the Gene Expression Omnibus (GEO) of the National Center for Biotechnology Information  under GEO Series record GSE12546.

### Visualization and classification of alternative splicingevents

Before validating the exon array data by various approaches, an expert investigation on gene structure and genomic context was carried out to assess the positions and surrounding mRNA/cDNA sequences of alternatively spliced exons. The Blat program [57] was used to map alternatively spliced exons in the UCSC Genome Browser  referred to the mRNA/cDNA sequences (from the NCBI RefSeq and GenBank databases) or expressed sequence tags (ESTs). Alternatively spliced multi-exon genes were classified into six splicing patterns according to the relative positions of the affected probe selected regions (PSRs) in exons and genes based on the sequence mapping. These classifications were cassette exons, namely exon inclusion and exon skipping, alternative promoters, alternative polyadenylation, alternative donor sites, alternative acceptor sites and intron retention.

### Function and pathway analysis

GO, protein function, and pathway enrichment analyses were carried out by the DAVID tool [58]. DEGs and alternatively spliced genes were mapped to the KEGG database using GenMAPP software, in order to visualize their distributions in the pathways [59].

After detecting alternatively spliced exons, their sequences and gene annotations were obtained from the Affymetrix website . The protein sequences of the coding regions of alternatively spliced exons were extracted from the NCBI RefSeq database by a in-house developed Perl program [60]. The InterProScan software was used to search protein domains via the interfaces of the PFAM, PROSITE, PRODOM, and SMART databases [61].

### Literature mining for functional modules

The purpose of the analysis is to find functional modules from complex biological networks. The functional module was defined as a part of a biological network with specific functions and topological features [62]. The nodes represent genes, and the links represent regulatory relationships between genes in the modules. A two-step literature mining strategy was performed on up- and downregulated genes to find activated functional modules in affected HUVECs. First, we used the cytoscape plugin "Agilent Literature Search" to construct the biological networks by a literature mining algorithm [63]. Only direct regulatory relationships between genes were preserved in building the network. Second, some orphan nodes and fake links were manually removed by checking relevant sentences in the obtained literatures in the first step. During the manual module check, the nodes were annotated by description from NCBI Entrez Gene [64], and the links were classified by regulatory relationships stated in the sentences from the relevant literature.

## Authors' contributions

XH carried out the exon array analysis, data interpretation and drafted the paper. PL carried out cell culture, sample preparation, cell apoptosis analysis and RT-PCR validation. YY, YG, YW and HL participated in cell apoptosis analysis and RT-PCR validation. ZFL, WQ, HZ and ZS participated in exon array analysis. MHD and ZS guided the project and proof-read the manuscript. CZ instigated, designed the study, supervised the analysis and finalized the manuscript. All authors read and approved the manuscript.

## Supplementary Material

Additional File 1 **List of upregulated and downregulated genes in hypoxia-treated HUVECs.** Upregulated genes are listed in sheet 1, and downregulated genes are listed in sheet 2. Click here for file

Additional File 2 **List of differentially expressed exons in hypoxia-treated HUVECs.** General inclusion events are listed in sheet 1 and general exon skipping events are listed in sheet 2. Overlaps between alternatively spliced genes and differentially expressed genes are listed in sheet 3. Click here for file

Additional File 3 **List of domains affected by alternative splicing.** Domains affected by alternative splicing are listed with their InterPro ID, names, alias, description, and literature, with functions relevant to hypoxia conditions. Click here for file

Additional File 4 **Figure S1 RT-PCR validation of selected DEGs.** The 18S rRNA was used as a control. The primer sequences are available in an online Excel table (See Additional file 5). Click here for file

Additional File 5 **Primer sequences for validating DEGs and alternative splicing events.** None. Click here for file

Additional File 6 **Annotation of nodes and edges in Figure**5. Functional descriptions of the "Node" (genes and proteins participated in the module) are listed in "Notes" column. The regulatory relationships between nodes (edges) are supported by literature citations. Click here for file

Additional File 7 **Evidence of combinational regulation between transcription and splicing.** Seventeen transcription factors undergoing alternative splicing are listed in sheet 1. Differentially expressed splicing factors (including SR proteins and hnRNP proteins) are listed in sheet 2. Click here for file
