# Supplementary figures and images for: Transcription and splicing regulation in human umbilical vein endothelial cells under hypoxic stress conditions by exon array
Source: BMC Genomics. 2009 Mar 25;10:126. doi: 10.1186/1471-2164-10-126 (PMC2678155; doi:10.1186/1471-2164-10-126)

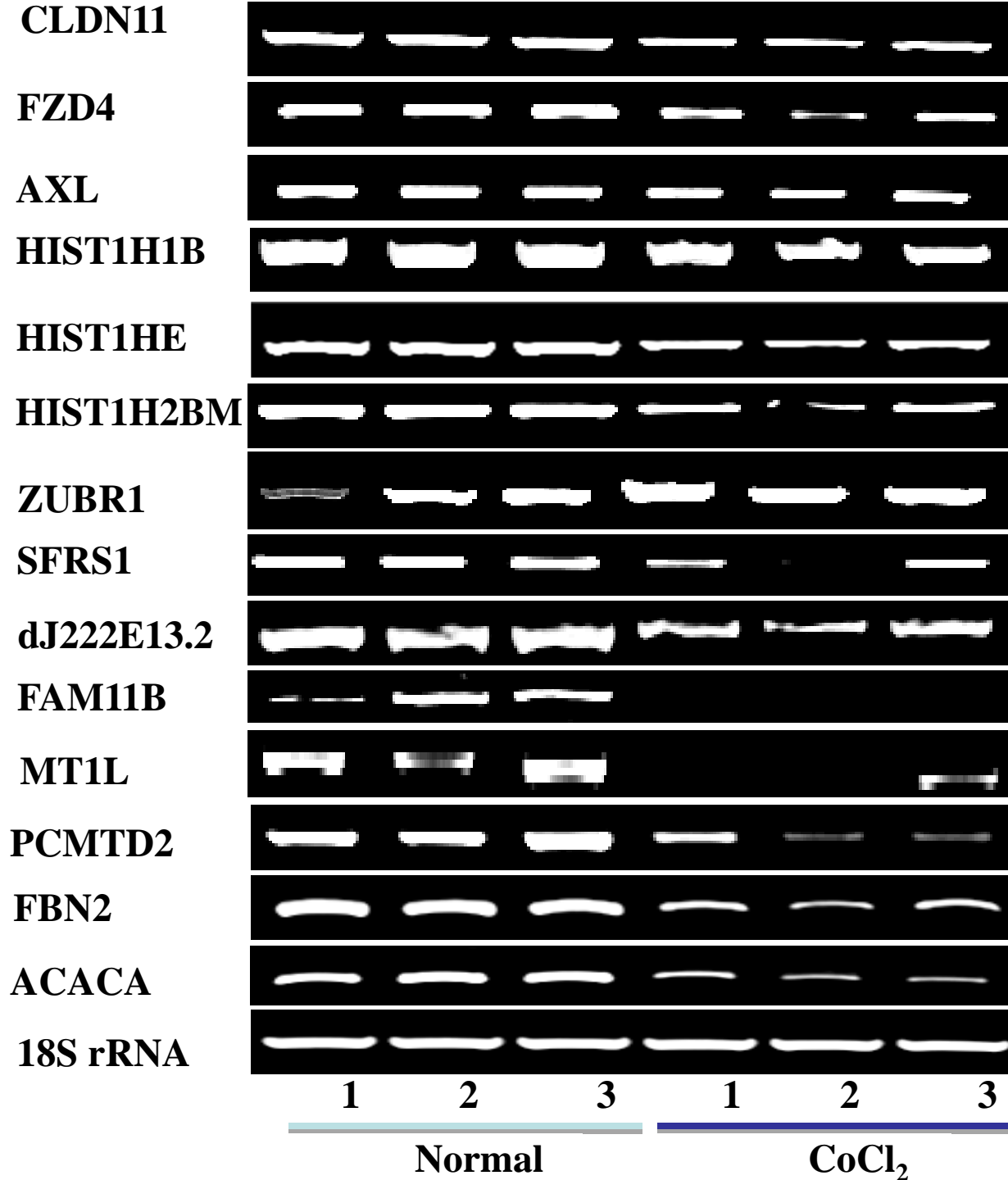

Supplement: Additional File 4 — Figure S1 RT-PCR validation of selected DEGs. The 18S rRNA was used as a control. The primer sequences are available in an online Excel table (See Additional file 5). [file 1471-2164-10-126-S4.pdf]
